# An Overlooked Perspective in Psychological Interventions to Reduce Anti-elderly Discriminatory Attitudes

**DOI:** 10.3389/fpsyg.2021.765394

**Published:** 2021-10-25

**Authors:** Yuho Shimizu

**Affiliations:** Graduate School of Humanities and Sociology, The University of Tokyo, Tokyo, Japan

**Keywords:** elderly people, discriminatory attitudes, stereotype embodiment theory, interventions, subjective time, intergenerational conflicts

## Introduction

The world's population is aging at a remarkable rate. The percentage of the world's population aged 65 and over was 5.1% in 1950, was 8.3% in 2015, and will increase to 17.8% by 2060 (United Nations, 2017). In this aging society, intergenerational conflicts between the elderly and the rest of the population are frequently observed in many workplaces and nursing care (Binstock, 2010). Anti-elderly discriminatory attitudes held by non-elderly people have been examined as one of the major causes of such intergenerational conflicts (North and Fiske, 2013). Specifically, the elderly is often perceived as incompetent and stubborn (McKenzie and Brown, 2014). Non-elderly people may also hold the discriminatory view that the elderly should pass down any resources, avoid excessive consumption of any shared social resources, and not behave as if they were younger (North and Fiske, 2013). These anti-elderly discriminatory attitudes lead to a decrease in the quality of life of the elderly (Levy et al., 2000), a disregard for the will of the elderly (Vitman et al., 2014), and an inhibition of the formation of a harmonious intergenerational society (Ishii and Tadooka, 2015). Based on the above, the affirmation of attitudes toward the elderly is an important issue in psychological research.

This opinion paper will begin with a broad overview of the interventions that have been implemented to reduce anti-elderly discriminatory attitudes. Then, an important perspective specific to a social group of the elderly, which have not been sufficiently paid attention to, is pointed out; all people will eventually belong to a social group of the elderly. As a theory that incorporates this perspective, Levy's (2009) stereotype embodiment theory (SET) will be introduced, and a typical factor (i.e., subjective time to become elderly) that should be focused on in future interventions to reduce anti-elderly discriminatory attitudes, will be discussed. In this paper, the fact that we all become elderly is focused on, and this mainly refers to healthy aging, not pathological aging.

## Previous Interventions to Combat the Anti-Elderly Discriminatory Attitudes

To reduce anti-elderly discriminatory attitudes, a wide range of strategies have been conducted. Previous research has conducted educational interventions which aim to demystify certain commonly misunderstood aspects of the aging and elderly (Chonody, 2015; Lytle and Levy, 2019). For example, Wurtele and Maruyama (2013) found that, after participants were presented with accurate information about the elderly, their anti-elderly discriminatory attitudes significantly decreased. The advantage of such educational interventions is that they are relatively easy to implement and can be delivered to a wide range of participants simultaneously. On the contrary, interventions to encourage perspective taking by participants' experience in the impaired physical movements of the elderly (Berthold et al., 2013) and experience in the elderly's appearance using virtual reality (Oh et al., 2016), are also shown to be effective. These types of interventions will continue to increase as scientific technology have developed in recent years. In addition, various studies aimed to reduce anti-elderly discriminatory attitudes through direct contact experience with the elderly (Allan and Johnson, 2008). For instance, Meshel and McGlynn (2004) conducted a 6-week intergenerational exchange program; elementary and junior high school students' attitudes toward the elderly became more positive after the intervention. This result is consistent with the classical findings on mere exposure effects (Zajonc, 1968; Kwan et al., 2015). Extended contact experiences, in which participants imagine getting positive and favorable contact experience with the elderly, are also effective in reducing discriminatory attitudes (Drury et al., 2016; Pekçetin et al., 2021). A strategy of extended contact has the advantage of being relatively easy to implement even in environments where it is difficult to get contact experience with the elderly.

Intervention strategies described above contribute to reducing anti-elderly discriminatory attitudes, but they miss an important perspective specific to a social group of the elderly; all people will eventually belong to a social group of the elderly. This characteristic does not apply to other contexts of prejudice, such as gender or race. Based on the above, it is important to incorporate the perspective that everyone will eventually become an elderly into the context of prejudice against the elderly, but there has been insufficient research to date (Levy, 2009; Takeuchi, 2016; Shimizu et al., 2021). Therefore, in this opinion paper, Levy's (2009) SET is focused on as a theory that incorporates this perspective.

## Stereotype Embodiment Theory

SET is a theory that discusses the effects of elderly stereotypes on the perceivers themselves. SET consists of four major processes: internalization, unconscious operation, salience gain from self-relevance, and utilization of multiple pathways (Levy, 2009). Internalization is the process by which people are faced with and internalize elderly stereotypes throughout their lives, and the process begins in childhood (Levy and Banaji, 2002). Unconscious operation is the process by which internalized stereotypes, as described above, automatically and unconsciously influence people's judgments and actions (Bargh et al., 1996). Salience gain from self-relevance is the process by which internalized stereotypes of the elderly are understood as highly self-relevant incidents and this is a process that we all go through as we get older (Levy, 2009). Utilization of multiple pathways is the process by which self-associated stereotypes of the elderly influence themselves psychologically, behaviorally, and physiologically (Levy et al., 2006; Wurm and Benyamini, 2014; Chasteen et al., 2015). For example, it has been shown that elderly people who have strongly internalized negative elderly stereotypes are more likely to feel stressed and lonely (McHugh, 2003).

SET is unique in that it discusses the effects of elderly stereotypes on perceivers themselves, focusing on the temporal dimension. Everyone will eventually become elderly, and those who have a negative view of the elderly when they are young are more likely to be affected by the undesirable effects described above (Levy, 2009). It is an inherent characteristic of the elderly that perceivers of stereotypes and prejudices will one day become members of the stigmatized group. Based on the above, it is necessary to incorporate this temporal dimension into the discussion in the context of reducing anti-elderly discriminatory attitudes. However, a major problem is that there has not been enough discussion that incorporates this perspective.

## Discussion

As strongly argued in the SET, the elderly is unique in which everyone eventually belongs to. In order to incorporate this perspective into the context of reducing anti-elderly discriminatory attitudes, it would be useful to focus on the subjective time to become elderly. It has been reported that there are large individual differences in people's sense of time (Jokic et al., 2018; Stam et al., 2020). In other words, even if the time is the same length, each person feels it in a different way. Specifically, some people believe that becoming elderly is not a long way off, despite their actual age being young, while others believe that becoming elderly is still a long way off, despite their actual age being relatively old.

Those who believe that becoming elderly is still a long way off will be more likely to perceive the elderly as separate from the self and unlikely to imagine themselves when they become elderly. Such cognition of separating the elderly from the self as temporally distant is noteworthy because it is likely to contribute to anti-elderly discriminatory attitudes and, in turn, to the reinforcement of intergenerational conflicts. From the perspective of social identity theory (Tajfel, 1981; Onorato and Turner, 2004), it can be said that when we perceive others as different and distant from ourselves, we perceive them as an outgroup and direct discriminatory attitudes toward them. Based on the above, interventions that make participants feel that the subjective time to become elderly is shorter, may be effective. However, in the context of reducing anti-elderly discriminatory attitudes, the impact of the subjective time to become elderly has not been sufficiently examined. Future research should explore interventions such as experimentally manipulating the subjective time to become elderly.

In this opinion paper, a broad overview of the interventions to reduce anti-elderly discriminatory attitudes is shown. An important perspective; all people will eventually belong to a social group of the elderly, has been overlooked in the literature. To incorporate such perspective, Levy's (2009) SET was introduced, and it would be effective to focus on the subjective time to become elderly. Future studies should investigate and focus on the temporal dimension to find more effective intervention methods. Therefore, the concept of subjective time to become elderly deserves a great deal of attention. This opinion paper will contribute to the direction of psychological intervention research and its future development.

## Author Contributions

YS: article development, composition, draft review, and creative oversight.

## Conflict of Interest

The author declares that the research was conducted in the absence of any commercial or financial relationships that could be construed as a potential conflict of interest.

## Publisher's Note

All claims expressed in this article are solely those of the authors and do not necessarily represent those of their affiliated organizations, or those of the publisher, the editors and the reviewers. Any product that may be evaluated in this article, or claim that may be made by its manufacturer, is not guaranteed or endorsed by the publisher.
